# Perception Matters: The Influence of School Ethnic Racial Context on Ethnic Racial Identity Development for Black Adolescents

**DOI:** 10.3390/bs14100872

**Published:** 2024-09-27

**Authors:** Amirah Saafir, Sandra Graham

**Affiliations:** 1 Child and Adolescent Studies Department, California State University, Fullerton, CA 92831, USA; 2 Department of Education, University of California, Los Angeles, CA 90095, USA; graham@gseis.ucla.edu

**Keywords:** ethnic–racial identity, African American, school context, adolescents, peer discrimination

## Abstract

In the current study, latent growth curve modeling is used to explore growth in ethnic–racial identity (ERI) commitment from 9th to 12th grade as a function of two aspects of the school ethnic–racial environment—perceived representation among peers and perceived discrimination from peers. The participants included 237 students that self-identified as African American (Mage = 14.7; 50% female). The results showed that perceiving more Black peers at school buffered the negative impact of racial discrimination from peers on ERI commitment. Further, the positive impact of perceived representation remained significant even after controlling for other markers of school ethnic–racial context including objective representation and school ethnic–racial diversity. The findings have implications for the environmental factors that support ERI development as well as how we study and conceptualize the influence of the school ethnic–racial environment.

## 1. Introduction

In an era where racial injustice remains deeply embedded in the fabric of American society, the psychological well-being of Black youth is more precarious than ever. Persistent manifestations of racism underscore the urgent need to understand and foster resilience among Black adolescents. While systemic change is essential, Margaret Beale Spencer’s Psychological Variant of the Ecological Systems Theory (PVEST) highlights the equally critical need to explore cultural resources that empower Black youth to navigate and resist the harmful effects of racialized stressors [1]. One such resource is a robust ethnic–racial identity (ERI), which encompasses a deep sense of connection and pride in one’s racial group membership [2]. ERI has been consistently linked to positive psychosocial outcomes for Black adolescents, serving as a buffer against the damaging effects of racial discrimination (e.g., [3]). Given the utility of ERI in supporting adolescent psychosocial well-being, in this paper, the contextual factors are that may support the development of a strong positive sense of ERI. Specifically, we explored the impact of perceived ethnic–racial representation at school on the development of ERI for Black high schoolers as well as the utility of same race peers in supporting ERI in the face of racial discrimination. 

### 1.1. Ethnic Racial Identity (ERI) and ERI Commitment

The research consistently shows that a strong, positive sense of ethnic/racial identity (ERI) is linked to positive adjustment outcomes, especially for Black youth (e.g., [3,4]). ERI is a multi-faceted concept that captures both a sense of connection to and beliefs about one’s ethnic–racial group membership [2]. Researchers have identified several distinct dimensions of ERI that capture both the content of an individual’s beliefs about their ethnic–racial group membership and the development of those beliefs [5,6,7,8].

In this study, we focused on how perceptions of the school’s ethnic–racial environment impact the development of ERI. Jean Phinney’s widely used model of ERI development emphasizes the process of exploring group membership (ERI exploration) and developing positive feelings and connection to the group (ERI commitment) [6]. The research has shown that these dimensions grow and change throughout adolescence [9] and especially when entering a new school context, such as starting high school [10]. Additionally, the ERI literature highlights the importance of examining the unique contributions and developmental patterns of its specific aspects (e.g., [5]).

As such, we chose to focus on ERI commitment specifically for two key reasons: its developmental nature and its emphasis on affect towards one’s ethnic–racial group. The research overwhelmingly demonstrates the positive developmental impact of having affirmative feelings about being Black. These positive feelings are associated with a range of beneficial outcomes, including fewer depressive symptoms, lower risk of substance abuse, improved academic achievement, and enhanced overall well-being [3,11,12,13].

Moreover, and particularly relevant to this study, positive feelings about being Black have been found to mitigate the negative psychological impact of racial discrimination (e.g., [4,14,15,16]). This suggests that ERI commitment may serve as a crucial tool of resilience for Black youth, especially when confronted with negative racialized stressors.

Given these insights, in our current study, we explore how perceptions of the school’s ethnic–racial environment either supports or hinders the development of ERI commitment. By focusing on this specific dimension of ethnic–racial identity, we aim to understand the factors that contribute to this important aspect of resilience in Black youth.

### 1.2. School Ethnic Racial Context

Ecological systems theory highlights the influence of proximal settings, like family and school, on child development [17]. Consistent with this theory, the school ethnic–racial environment plays an important role in how adolescents learn about and make sense of their ethnic–racial group membership [18,19,20,21]. School ethnic–racial context is multifaceted, including not only the ethnic–racial composition of schools but also the attitudes about racial and ethnic diversity held by teachers, peers, and administrators. Further, individual experiences and perceptions of the school ethnic–racial environment can vary significantly, as the research shows that the overall school context does not always match students’ daily experiences [22]. Therefore, an approach that centers individualized student experiences with the school ethnic–racial environment is necessary to account for students’ perceptions based on their day-to-day experiences [23]. As such, the current study focuses on students’ perceptions of the school ethnic–racial environment in order to capture their unique experience.

### 1.3. Representation, Discrimination and ERI Commitment

Specifically, we were interested in how perceived representation and perceived discrimination from peers influence the development of ERI commitment in high school. Representation, or having access to same-race peers at school, is especially beneficial for Black students in developing a sense of belonging and exploring cultural identity. For instance, among a sample of Black high school students, having more Black friends was associated with more positive feelings about their racial group membership [24]. Additionally, having more contact with Black peers at school is linked to greater stability in ERI over time [25] and an increased sense of the importance of their ethnic–racial group membership [26]. 

Perceived racial discrimination, or the frequency with which students feel they are treated unfairly by peers due to their race, has also been linked to ERI—albeit in a complex manner. The theories of ERI development suggest that experiencing racial discrimination may serve as a catalyst for exploring one’s ERI and, thus, increasing the salience or importance of one’s ERI to their overall self-concept. However, the experiences of racial discrimination can also undermine the affective component of ERI development by fostering feelings of exclusion and marginalization [7,27]. For example, experiencing school-based racial discrimination has been found to be associated with feeling that one’s racial ethnic group is negatively perceived by the broader society [28]. Further, it has even been found that for Black adolescents, increased perceptions of racial discrimination from peers were associated with less positive feelings about being Black [29,30] and less of a sense of connection to other Black peers [29].

### 1.4. The Present Study

In the current study, the interplay between perceived representation and perceived discrimination is explored in its influence on the development of ERI commitment for Black high school students. To complete this study, we recruited a large, diverse sample of students from 26 middle schools in Northern and Southern California and followed them from 6th grade through the transition to 211 high schools, all of which varied in ethnic–racial composition. Consistent with the studies demonstrating the negative impact of racial discrimination on feelings about being Black [7,27], we hypothesized that the perceived discrimination from peers would negatively affect the growth in ERI commitment. However, given the evidence supporting the beneficial effects of having same-race peers on ERI [24,25,26], we further hypothesized that perceived representation would buffer the negative impact of discrimination, with perceiving more Black peers acting as a protective factor against the harmful effects of racial discrimination. Additionally, we investigated whether these individualized markers of the school context would remain significant even after controlling for objective markers, such as the percentage of same-race peers at school and overall school ethnic–racial diversity. This study contributes to the growing body of literature by simultaneously examining the impact of multiple aspects of the school ethnic environment on ERI development.

## 2. Methods

### 2.1. Participants

The data for this study come from a longitudinal study that examines the impact of ethnic–racial diversity on students’ psychosocial and educational outcomes from 6th grade to one year post high school. The total sample included 5991 students who were recruited from 26 middle schools in Northern and Southern California and followed until one year post high school. Approval from the university IRB board was obtained to complete this study.

To determine ethnicity/race, participants were asked to select from a list of 13 options. The ethnic composition of the original sample was 11% Black/African American, 15% East/Southeast Asian, 24% European American/White, 33% Latino/Mexican, 2% South Asian, 3% Filipino/Pacific Islander, 3% Middle Eastern, and <1% Native American. The analytic sample for this study is a subset of 237 students who self-identified as African American/Black (50% female; Mage = 14.7, SD = 0.38 in 9th grade) and had complete data on key study variables for a minimum of 3 out of the 4 time points.

### 2.2. Procedure

Participants were recruited in middle school in three cohorts from 2009 to 2011 and then re-recruited in 9th grade from the 211 high schools to which they transitioned. All students required parental consent and student assent to participate. Data for the current analysis were collected from Wave 5 to Wave 8 (spring of 9th grade to spring of 12th grade). Students completed surveys on iPads during non-academic classes while trained graduate student researchers monitored and aided as needed. Upon completion of the survey, students were thanked and given an honorarium.

### 2.3. Measures

#### 2.3.1. Outcomes

ERI Commitment 9th–12th Grade. Commitment was measured using a subset of 3 questions from the Multi-group Ethnic Identity Measure [6]. Respondents rated items (e.g., “I am proud that I am a member of my ethnic group”) using a 5-point Likert Scale; (1 = Definitely Yes 5 = Definitely No; reverse coded). The Cronbach alphas ranged from 0.78 to 0.87 across all four time points.

#### 2.3.2. Predictors

Perceived Same Race. Perceived same race was defined as the proportion of Black peers students perceive at their school. Perceived same race was measured each year using a single item, “How many students at this school are from your ethnic group?” Participants responded using a 7 pt Likert scale (1 = none or hardly any (less than 10%) to 7 = all or almost all (90–100%). In this study, we included perceived same race in 9th and 10th grade. Average perceived same race scores were 3.73 and 3.49 in 9th and 10th grade, respectively.

Peer Racial Discrimination. Racial discrimination was assessed using a measure of student racial discrimination. The measure was adapted from a version of the Adolescent Discrimination Distress Index (ADDI) [31]. Respondents rated 4 items (e.g., “Treated disrespectfully by other kids because of your race/ethnic group”) on a 5-point scale from 1 = Never to 5 = A Whole Lot (α = 0.83). The measures of school context for this study center the ethnic–racial makeup of the student body. As such, discrimination from peers (as opposed to teachers or other adults) was most relevant for this study. We included peer racial discrimination in 9th and 10th grade in this study. The Cronbach alpha was 0.78 in 9th grade and 0.79 in 10th grade.

#### 2.3.3. Covariates

Percent Same Race. Percent same race was defined as the proportion of Black students in a particular grade at each school out of the total number of students in that grade. This value was calculated separately for each school and was calculated using public data from the CDE. Percent same race for this sample ranged from 0.00 to 0.77 across time points.

School Diversity. School diversity was measured using CDE data to calculate Simpson’s index of diversity [32] at the grade level. The Simpson’s diversity index reflects the probability that two students randomly selected from a group (i.e., ninth grade) will belong to different ethnic groups. Values range from 0 to 1 with higher values indicating more school diversity. Simpson’s scores for this sample range from 0.03 to 0.77 across time points.

$$D_{C}=1-\sum_{i=1}^{g}{P_{i}}^{2}$$

Proportion Free and Reduced Lunch. School SES was measured using proportion of students receiving free or reduced-price lunch (FRL) as a proxy. This value was calculated using CDE data. In our sample, FRL ranged from 0.05 to 0.97 in high school. 

Sex. Sex was measured using self-report at Wave 5 (spring of 9th grade). It is important to note that at Wave 5, the options included on the survey for this item were dichotomous such that students had to pick male or female. A total of 50% of the analytic sample identified as female. We also included the following:

Parent Education. Socioeconomic status was measured using parent education levels as a proxy. Parents/guardians were asked to complete a brief survey at the beginning of 6th grade and the beginning of 9th grade at the time of consent. For this study, we used the reported education level for 6th grade. Included in this survey was a question on parent educational background where parents were asked to indicate their highest education level from the following options: (1) elementary/junior high; (2) some high school; (3) high school diploma or equivalent; (4) some college; (5) 4-year college degree; or (6) graduate degree (M = 4.23, SD = 1.10).

## 3. Results

### 3.1. Analytic Plan

Preliminary analyses examined the descriptive properties and bivariate correlations among the main study variables (Appendix A). We first estimated an unconditional multi-level latent growth curve to model growth in the ERI commitment from 9th to 12th grade. Next, we developed a conditional model to explore the impact of perceived representation and peer racial discrimination on the development of commitment. Specifically, we estimated the impact of perceived representation in 10th grade on the growth in ERI commitment from 9th to 12th grade, controlling for 9th grade perceived representation. Additionally, although our main analysis focuses on perceptions of the school ethnic–racial context, we also controlled for objective markers of Black student representation (percent same-race peers) and school ethnic–racial diversity. Recognizing the connections between race, gender, and socioeconomic status, we included measures of gender and SES at both the individual and school levels.

All analyses were conducted in Mplus version 7.3 [33]. As with most longitudinal studies, not all participants had complete data at each assessment wave. The estimation procedure maximum likelihood with robust standard errors (MLR) was specified to handle missing data through full-information maximum likelihood (FIML). FIML allows for the inclusion of all available data in the analyses by fitting the covariance structure model directly to the observed raw data for each participant [34]. 

In order to ensure that missing data were handled appropriately, we conducted Little’s MCAR test, which indicated that the data were missing completely at random (*p* = 0.46; MCAR), confirming that there was no systematic pattern in the missingness. This finding supports the use of FIML as the most appropriate method for handling the missing data in our study. Additionally, to examine whether participants with missing data differed significantly from those who completed all waves, we conducted a series of chi-squared tests on key demographic variables. The tests revealed no significant differences in parent education level (*p* = 0.10), suggesting that missingness did not systematically differ by this variable. However, we did find a marginally significant difference in gender, with slightly more boys missing data (N = 102) compared to girls (N = 95), *p* = 0.02. This potential gender difference was considered in our interpretation of the results.

The final analytic sample consisted of 237 Black participants. Reports of the number of participants for each measure at each data wave can be found in Appendix A. We used TYPE = COMPLEX to account for the nesting of students within schools [34]. 

### 3.2. Growth in ERI Commitment from 9th to 12th Grade

**Figure 1:**
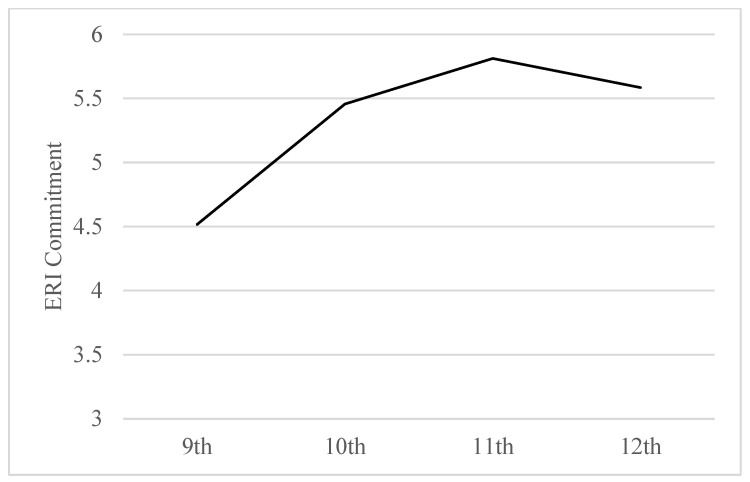
Unconditional growth model predicting ERI commitment from 9th to 12th Grade.

Based on previous studies modeling growth patterns of ERI commitment, we expected a non-linear growth pattern, where the rate and nature of change in ERI commitment would vary throughout high school [35,36]. Our findings supported this expectation, showing that a quadratic equation was the best fit for our unconditional model. Specifically, ERI commitment grew rapidly from 9th to 10th grade and then began to attenuate in the 11th and 12th grade (Figure 1).

### 3.3. Impact of Perceived Representation and Peer Racial Discrimination

We then tested our conditional model to explore the impact of perceived representation and peer discrimination on change in ERI commitment. Model fit statistics indicated the conditional model fit the data well, with a CFI value of 0.97 and an RMSEA value of 0.04, which meet the established criteria for good model fit [37] (Table 1). The analyses revealed a significant main effect of peer racial discrimination on the growth of ERI commitment from 9th to 12th grade. More perceived racial discrimination from peers in 9th grade was associated with less ERI commitment at the start of high school (b = −0.24; *p* < 0.01). Additionally, perceiving more racial discrimination from peers in 10th grade was linked to slower growth in ERI commitment throughout high school (b = −0.52; *p* < 0.01). These findings remained significant even after adding our objective contextual markers to the model—percent same race peers and school diversity (Table 2).

**Figure 2:**
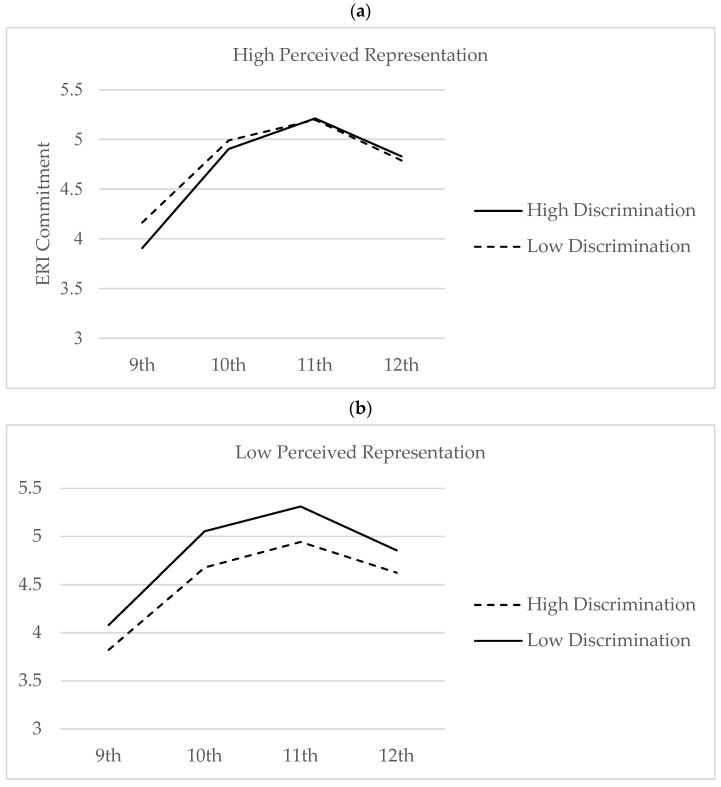
(**a**,**b**) Change in ERI commitment from 9th to 12th Grade as a function of perceived representation and peer discrimination.

While there was no significant main effect of perceived representation on ERI commitment in 9th grade, there was a significant impact in 10th grade. Perceiving more Black peers in 10th grade was associated with a slight decrease in ERI commitment over time (b = −0.18; *p* < 0.05). However, a significant interaction was found between perceived representation and peer racial discrimination on the growth in ERI commitment (b = 0.13; *p* < 0.05; Table 2). The effect size for this finding, according to Cohen’s d, ranged from 0.2 to 0.5, indicating a moderate effect size. Graphing this finding revealed that in the face of racial discrimination from peers, Black high school students who perceived more Black peers fared better on commitment than those who perceived fewer Black peers (Figure 2). This interaction also remained significant after controlling for objective markers of school context (Table 2).

## 4. Discussion

The purpose of this study was to understand how individualized perceptions of the school’s ethnic–racial environment impact on ethnic–racial identity (ERI) development. We specifically wanted to understand how perceptions of representation and peer racial discrimination impacted growth in ERI commitment. We chose to focus on ERI commitment because it captures the positive feelings and sense of connection to one’s ethnic–racial group, which research suggests plays a critical role in resilience and positive adjustment, particularly in the face of discrimination. Consistent with our hypothesis, findings indicated that perceiving more discrimination from peers at school predicted less growth in ERI commitment from 9th to 12th grade. However, this negative effect was mitigated when perceived representation of Black peers was high. This buffering effect of perceived representation remained significant even after controlling for the actual ethnic–racial composition of the school. These findings underscore the importance of school contextual factors in impacting positive ERI commitment development, with perceived representation playing a key role in promoting growth and buffering against the negative impacts of racialized environmental stressors on ERI commitment.

Our findings align with the work of Juan Del Toro and other scholars who have explored the connections between various aspects of the school ethnic–racial environment and ERI development. Del Toro and Wang, for example, found that perceiving positive cultural socialization within the school setting was associated with greater ERI exploration and commitment among African American high school students [38]. Importantly, their work demonstrated that ERI commitment mediated the relationship between cultural socialization and academic achievement, meaning that students who experienced stronger cultural socialization showed higher ERI commitment after one year, which subsequently contributed to improved academic performance. These findings highlight the tangible benefits of a school environment that fosters positive identity development for Black adolescents’ overall success. In relation to our study, it seems perceived representation of same-race peers may similarly contribute to Black students’ perceptions of a supportive school climate.

Moreover, our results show that the presence of more same-race peers becomes especially critical when Black youth face racial discrimination from peers. Mims and Williams [39] offer an essential framework for understanding the interplay between representation, discrimination, and ERI development. Specifically, they emphasize that Black youth, especially girls, interpret their ERI through the lens of the stereotypical messages they encounter in school settings. This aligns with Hughes et al. [20], who argue that ethnic–racial socialization experiences, discrimination, and ERI are interconnected, with the effect of one depending on the presence of the others. Our findings extend these arguments by demonstrating that same-race peer representation plays a critical role in helping Black youth navigate and interpret racialized messages within schools and peer groups.

When analyzed through the lens of phenomenological variant of ecological systems theory (PVEST), a complex interplay of risk and protective factors emerges in the identity development of Black youth. Perceived racial discrimination from peers functions as a significant risk factor, negatively impacting ERI commitment and illustrating how racial stressors within the school environment can influence developmental pathways. In contrast, the perceived representation of Black peers serves as a protective factor, buffering the effects of these stressors. This interaction reinforces PVEST’s core idea that environmental factors—both challenging and supportive—shape the evolving self-concept of Black youth in meaningful ways.

Our findings also emphasize the importance of considering students’ experience-focused aspects of the school ethnic–racial environment. Notably, the positive effect of perceived representation on ERI commitment persisted even after accounting for school diversity and the percentage of same-race peers. This suggests that the subjective context—students’ unique experiences with the school’s ethnic–racial environment—plays a distinct role in influencing developmental outcomes. This idea is supported by scholars like Christy Byrd, who emphasize the importance of and individualized experience-focused approach to understanding school contextual factors on adolescent development [23].

The research indicates that the overall ethnic–racial context of a school does not always align with the context students experience daily [22]. Therefore, understanding subjective experiences of the school context can be as explanatory as objective markers [9]. Subjective and objective measures do not always align and may have different influences. For example, correlation analyses from our study revealed a moderate, significant relationship between perceived and objective representation (r = 0.64; *p* < 0.05; Appendix A). This pattern shows some alignment but also suggests a mismatch, indicating that subjective and objective contexts may function differently.

The existing literature also suggests that different measures of context are associated with different outcomes. For example, a recent study by Conway-Turner and colleagues explored how three measures of school ethnic–racial composition—school diversity, minority concentration (proportion of non-White students), and percent same-race peers—impacted GPA and test scores for elementary students [40]. For Black students, they found a negative relationship between school diversity and academic outcomes, a positive relationship between minority concentration and academic outcomes, and no relationship between percent same-race peers and academic outcomes. These findings illustrate that even within objective markers, the impact on student outcomes varies. However, few studies have concurrently examined multiple markers of school context, especially considering both objective and subjective markers.

Our study also suggests that the impact of the school ethnic–racial context on psychosocial well-being varies based on the relationships between different aspects of the school environment. For instance, we found that the relationship between racial discrimination and ERI commitment depended on perceived representation. Similarly, Byrd and Chavous found that the benefit of private regard (positive feelings about being Black) in supporting intrinsic motivation among African American 11th graders was dependent on perceiving a positive school ethnic–racial climate [41]. In a more recent study, it was found for Black middle and high school students that perceptions of the overall school climate were influenced by perceptions of support for cultural diversity at school [42], and this relationship was mediated by ERI. This interplay between different aspects of the school environment is important not just for Black students. For example, a study of Latinx high schoolers in California found that students reported better math attitudes when they felt represented in their math classes and perceived a positive overall racial climate at school [43]. These findings highlight the need for more studies considering the multidimensional nature of the school ethnic–racial environment.

### Limitations and Future Directions

While the findings of this study are significant, it is important to acknowledge its limitations. We focused on ERI commitment due to its strong connection with positive outcomes for Black youth. However, ERI is a multidimensional construct, and the buffering effect of perceived representation might not extend to other dimensions of ERI. For instance, how does perceived representation affect perceptions of outgroup opinions about Black people or the salience of being Black? Is increased perceived representation most beneficial for these ERI aspects, or might other elements of the school ethnic–racial environment play a more crucial role?

Another limitation is the sample size. Although reasonable, it was insufficient to explore all the nuanced interactions between different contextual factors. For example, how do perceived representation of Black peers, the percentage of Black peers, and overall school diversity interact to influence ERI commitment? The future research should address these questions using larger, longitudinal samples of Black students.

## 5. Conclusions

The current study has significant implications for our approach to diversity, equity, and inclusion (DEI) efforts. While quantitative metrics are important, achieving true equity is not just about numbers. Effective DEI initiatives must go beyond mere statistics to address the actual day-to-day experiences of students within these environments. This study highlights that representation and the feeling of being represented are not always the same. Just as students cannot fully benefit from diversity if they do not encounter diverse peers in their classes [44], similarly, the impact of representation extends beyond mere presence.

This distinction is particularly crucial for understanding the experiences of Black students, who often perceive the school ethnic–racial environment less positively than their peers of other races [42]. Future research should delve into why subjective and objective representations differ and identify the most influential factors in shaping students’ perceptions of their school environment. Are these perceptions influenced by the subspaces students inhabit, such as their classes, friend groups, or extracurricular activities?

By understanding the predictors of students’ perceptions, we can approach school diversity and representation in a nuanced manner. This approach should prioritize not just the numerical presence but also the quality of students’ day-to-day experiences, especially for Black students. Examining the school ethnic–racial environment through a multidimensional, individualized lens will enable schools and policymakers to target specific features of the environment to support specific developmental outcomes.

## Figures and Tables

**Table 1:** Model fit statistics for growth model of ERI commitment from 9 to 12th Grade.

Model	CFI	RMSEA	Inter	Slope	Quad
Unconditional	0.99	0.03	4.31 ***	0.07 *	−0.01 *
Conditional	0.97	0.04	4.20 ***	1.85 **	−0.50 **

Note. *p* < 0.05 *, *p* < 0.01 **, *p* < 0.001 ***.

**Table 2:** Impact of perceived representation and peer racial discrimination on ERI commitment from 9th to 12th Grade.

Variable	Intercept (SE)	β	Slope (SE)	β	Quadratic (SE)	β
**Means**	4.20 (0.35) ***	7.64	1.85 (0.53) **	6.20	−0.50 (0.18) **	−4.96
**Perceived Rep**						
9th perceived same	0.03 (0.03)	0.08	0.01 (0.04)	0.06	−0.01 (0.02)	−0.16
10th perceived same	––	––	−0.18 (0.08) *	−0.80	0.05 (0.03)	0.56
**Peer Discrim**						
9th peer discrim	−0.24 (0.07) **	−0.22	0.07 (0.04)	−0.13	0.05 (0.03)	0.25
10th peer discrim	––	––	−0.52 (0.16) **	−0.71	0.13 (0.06) *	0.47
**Interaction**						
10th Rep × discrim	––	––	0.13 (0.05) **	1.04	−0.03 (0.02) *	−0.75
**Covariates**						
gender	−0.09 (0.08)	−0.08	−0.11 (0.09)	−0.18	0.07 (0.03) *	0.32
Parent edu	−0.01 (0.04)	−0.02	−0.07 (0.04)	−0.24	0.02 (0.01)	0.17

Note: Time varying covariates (percent same race peers, school diversity and free and reduced lunch) were included in the model and were non-significant; *p* < 0.05 *, *p* < 0.01 **, *p* < 0.001 ***.

## Data Availability

The datasets presented in this article are not readily available in the interest of the privacy of participants. Requests to access the datasets should be directed to [n.kline@ucla.edu].

## Appendix A

**Table A1:** Intercorrelations and descriptive statistics for main study variables.

Variable	1	2	3	4	5	6	7	8	9	10	11	12
1. Commitment 9th	-											
2. Commitment 10th	0.61 **	-										
3. Commitment 11th	0.41 **	0.56 **	-									
4. Commitment 12th	0.37 **	0.45 **	0.56 **	-								
5. Perceived Same 9th	0.11 *	0.12 *	0.08	0.02	-							
6. Perceived Same 10th	0.12 *	0.09	0.08	0.03	0.72 **	-						
7. Racial Discrimination 9th	−0.19 **	−0.18 **	−0.13 *	−0.05	−0.14 **	−0.12 *	-					
8. Racial Discrimination 10th	−0.19 **	−0.17 **	−0.19 **	−0.16 **	−0.13 *	−0.16 **	0.55 **	-				
9. Percent Same Race 9th	0.10	−0.04	0.05	0.04	0.44 **	0.51 **	−0.06	0.02	-			
10. Percent Same Race 10th	0.09	0.00	0.03	0.02	0.57 **	0.64 **	−0.16 **	−0.08	0.79 **	-		
11. Diversity 9th	−0.04	−0.01	−0.02	−0.10	−0.12 *	−0.18 **	0.06	0.04	−0.26 **	−0.38 **	-	
12. Diversity 10th	−0.11	−0.08	−0.03	−0.10	−0.15 *	−0.19 **	−0.09	0.01	−0.31 **	−0.39 **	0.77 **	-
N	397	424	378	384	396	419	392	416	388	412	388	412
Mean	4.34	4.37	4.43	4.45	3.73	3.49	1.34	1.33	0.24	0.25	0.59	0.60
Std Deviation	0.62	0.63	0.70	0.68	1.4	0.22	0.54	0.53	0.21	0.20	0.15	0.13

Note. *p* < 0.05 *, *p* < 0.01 **.
